# Serum Concentration of Ropivacaine After Repeated Administration to Several Parts of the Head During Awake Craniotomy: A Prospective Cohort Study

**DOI:** 10.3389/fmed.2022.834334

**Published:** 2022-05-04

**Authors:** Takehito Sato, Takahiro Ando, Ichiko Asano, Atsushi Mori, Kazuya Motomura, Kimitoshi Nishiwaki

**Affiliations:** ^1^Nagoya University Hospital Department of Anesthesiology, Nagoya, Japan; ^2^Department of Perioperative Management System, Nagoya University Graduate School of Medicine, Nagoya, Japan; ^3^Department of Neurosurgery, Nagoya University Graduate School of Medicine, Nagoya, Japan

**Keywords:** awake craniotomy, ropivacaine anesthetics, scalp block, anesthesiology, local anesthesia

## Abstract

**Introduction:**

During awake craniotomy, effective use of local anesthetics, such as ropivacaine, is critical. Blood concentrations of ropivacaine after repeated administration over a short period during awake craniotomy have not been studied.

**Materials and Methods:**

In this prospective cohort study, we evaluated serum concentrations of ropivacaine 15 min after each administration during awake craniotomy at Nagoya University Hospital between April 5, 2018 and August 31, 2019 to determine the safe dose. A total of 30 patients scheduled to undergo elective awake craniotomy were included. Patients were injected with 0.375% ropivacaine before the awake phase at the following points: scalp block (T1), headpin area (T2), skin incision area (T3), temporal muscle (T4), and dura mater (T5). Arterial blood samples were collected 15 min after ropivacaine administration. In addition to the blood concentrations of ropivacaine, complications during the awake phase were evaluated as secondary endpoints.

**Results:**

The mean total dose of ropivacaine was 5.01 ± 0.68 mg/kg (maximum total dose: 6.30 mg/kg). The mean interval from T1 to T5 was 128.0 ± 17.7 min. The maximum serum concentration did not exceed the toxicity threshold of 4.3 μg/mL in any patient (mean serum concentration: T1, 1.23 ± 0.36 μg/mL; T5, 0.82 ± 0.26 μg/mL). No addiction symptoms were observed during awakening in any case.

**Conclusion:**

Our results show that, in cases of awake craniotomy with repeated anesthetic administration, a total dose of up to 5.0 mg/kg is safe, without addiction symptoms. Relatively large amounts of ropivacaine can be safely injected during awake craniotomy.

## Introduction

Awake craniotomy (AC) is a novel method for the resection of eloquent brain tumors to minimize neurological complications (1, 2). Patients need to be awake during the operation, and analgesic and local anesthetic administration are essential at the scalp block, headpin insertion site, surgical site, and temporal muscle and dura mater to ensure a safe operation (3–5). Ropivacaine has been widely used as a local anesthetic agent for AC (6, 7), and large amounts of local anesthetics are often used intraoperatively (5). The maximum safe dose of ropivacaine for AC is 3.60 mg/kg.

It is important to use local anesthetics appropriately during AC. Inadequate administration of local anesthetics can cause pain and make AC difficult (8), while overdose can lead to local anesthetic systemic intoxication. To reduce this risk, appropriate dosage and blood concentrations must be considered. Few reports have quantified the serum concentration of ropivacaine, which is repeatedly administered before the awake phase of surgery (6, 7). Moreover, there are no reports on the blood concentrations of ropivacaine during AC after repeated administration over a short period. In this study, we measured the serum concentration of ropivacaine after repeated administration to several parts of the head during AC, to determine whether ropivacaine had reached the toxic plasma level and to determine its safe intraoperative dose.

## Materials and Methods

### Ethics Approval Statement

All procedures performed in studies involving human participants were in accordance with the ethical standards of the institutional and/or national research committee and with the 1964 Declaration of Helsinki and its later amendments or comparable ethical standards. The study design was approved by the ethics committee of Nagoya University Hospital (approval number: 2017–0529; date of approval: April 5, 2018). The study was registered with the University Hospital Medical Information Network Clinical Trials Registry (UMIN000030896; date of approval: January 19, 2018). Written informed consent was obtained from all patients.

### Patients

Patients scheduled to undergo elective AC between April 5, 2018 and August 31, 2019 were included. Exclusion criteria were American Society of Anesthesiologists classification >3, liver failure (Child–Pugh classification B or C), severe respiratory diseases (severe obstructive pulmonary disease and severe restrictive lung disease), and patient refusal. Sample size was determined with reference to past reports (6, 7), and a total of 31 patients were included.

### Anesthesia

All patients underwent general anesthesia with scalp block, which was performed bilaterally. We performed scalp block as described previously (3). Ropivacaine (0.375%) with epinephrine (1:200,000) was used for the scalp block (T1). We tested the block effect using the cold test. If the block effect was insufficient, an additional dose of ropivacaine was administered (T1.5). Local anesthetic agents were used at the headpin site (T2: 10.0 mL), 0.375% ropivacaine with epinephrine (1:100,000) at the surgical site (T3: 10.0 mL), 0.375% ropivacaine with epinephrine (1:200,000) in the temporal muscle (T4: 15.0 mL), and 0.375% ropivacaine without epinephrine in the dura mater (T5: 0.5–1.0 mL).

We performed scalp blocks (supraorbital nerve and supratrochlear nerve, greater and lesser occipital nerve, auriculotemporal nerve, and zygomaticotemporal branch nerve blocks) before the sleep phase (5, 9). When performing scalp blocks, patients were administered low-dose fentanyl (50.0–150.0 μg) for analgesia during blocking.

A cannula was inserted into the radial artery for continuous measurement of arterial blood pressure and collection of blood samples. We monitored body temperature, oxygen saturation, and non-invasive blood pressure, and recorded the electrocardiogram. A urinary catheter was inserted.

General anesthesia was maintained with intravenous propofol (1% Diprivan Injection-kit® Aspen Japan, Tokyo, Japan), using a target control infusion system, with fentanyl and remifentanil. Supra-epiglottic devices were inserted in patients for airway management (i-gel® Intersurgical, Wokingham, United Kingdom) and bispectral index (between 40 and 60) monitoring was established. Before the awake phase, ropivacaine was administered to the headpin site (T2) and surgical site (T3). In all patients, mannitol and low-dose dexamethasone (6.6 mg) were introduced intravenously.

Once the neurosurgeon (K.M.) had administered ropivacaine to the temporal muscle (T4) and dura mater (T5) and could visualize the brain tumor, anesthetic drug infusion was stopped. After the patient woke up and could respond to verbal communication, we removed the supraglottic airway and the awake phase began. Metoclopramide (10.0 mg) or dexamethasone (6.6 mg) was administered intravenously if the patient complained of nausea. Furthermore, if patients complained of pain, local anesthetic (1.0% lidocaine with epinephrine 1:100,000) was injected into these sites, and flurbiprofen (50.0 mg) or low-dose fentanyl (25 μg) was administered intravenously for analgesia.

After tumor resection, the patients underwent intraoperative magnetic resonance imaging followed by induction of general anesthesia. The supraglottic airway was reinserted using a lateral caudal approach. At the end of the operation, patients were awakened from anesthesia and transferred to the intensive care unit. The anesthesia and the surgery were always performed by the same doctors (T.S. and K.M.) in all AC cases.

We recorded the patients' intraoperative data, such as age, sex, height, weight, body mass index, total amount of ropivacaine received, and any complications during the awake phase (hypertension, nausea, vomiting, seizure, and agitation). Local anesthetic intoxication was defined as complaints of dysgeusia, facial tingling, speech alteration, seizure, or inappropriate arrhythmia during the awake phase.

Intraoperative pain was classified as complaints of headache during the awake phase (numeric rating scale: >4) and use of analgesic agents (local anesthetic, flurbiprofen, or fentanyl). In previous studies, we defined hypertension as elevated blood pressure (>20% from baseline) or a systolic blood pressure >150 mmHg (10, 11).

After all scalp blocks were performed before anesthesia (paranesthesia) (5), we compared the block failures on each side using the cold test. The primary outcome was whether the arterial blood concentrations of ropivacaine exceeded the range of intoxication (4.3 μg/mL) (12), when administered at a total dose of 3.6 mg/kg, as reported previously (7). The secondary aim was to investigate complications during the awake phase, such as hypertension, nausea, vomiting, seizure, airway complications (insufficient ventilation), local anesthetic intoxication, hypoxia (oxygen saturation, <89%) (13), and agitation.

### Blood Collection

Patients were injected with 0.375% ropivacaine before the awake phase, and blood was collected 15 min after each administration as follows: scalp block (T1), headpin area (T2), skin incision area (T3), temporal muscle (T4), and dura mater (T5). If scalp blocks had only weak effects, we performed the blocks again. Ropivacaine was administered in a single dose not exceeding 3.0 mg/kg at each point.

Blood samples were collected in vacuum blood collection tubes containing a gel for serum separation (VENOJECT II® Terumo, Tokyo, Japan). The tubes were centrifuged for 15 min at 1,600 *g*. All serum samples were stored at −80°C until analysis. Drug-free serum samples were obtained from healthy volunteers.

### Measurement of Serum Ropivacaine Concentrations

#### Chemicals and Reagents

The primary standard solution of ropivacaine hydrochloride monohydrate was obtained from Toronto Research Chemicals (North York, Canada). The internal standard (IS), bupivacaine hydrochloride monohydrate, was purchased from Sigma-Aldrich (St. Louis, MO, USA), and all other chemicals, including high-performance liquid chromatography (HPLC)-grade acetonitrile, were purchased from Wako Pure Chemical Industries (Osaka, Japan). Water for the preparation of the mobile phase, standard solution, and IS solution was purified using an in-house Milli-Q system (Merck Millipore, Darmstadt, Germany).

#### Standard Sample Preparation

The primary stock solutions of ropivacaine and bupivacaine (5.0 and 10.0 mg/mL, respectively) were stored at −80°C until use. The secondary working solutions were prepared by serial dilution with water from the primary stock solution. Calibration standards were prepared using drug-free serum to make the final ropivacaine concentration range of 0.0–5.0 μg/mL.

#### Sample Preparation and Extraction

In a conical glass tube, IS solution (final concentration: 5.0 μg/mL) and 50.0 μL of 0.5 M potassium hydroxide were added to 500.0 μL of serum sample. After agitating the mixture, the serum sample was treated with 2.0 mL ethyl acetate to extract ropivacaine and the IS. The mixture was agitated for 2 min and centrifuged at 1,600 *g* for 15 min. The upper organic phase was transferred to another tube, and the serum sample was treated with 2.0 mL ethyl acetate. The upper organic phase was transferred to another tube. The combined organic phase was evaporated to dryness at 40°C (centrifugal concentrator, CC-105; TOMY SEIKO, Tokyo, Japan). The residue was dissolved in 200.0 μL of the mobile phase solvent and 50.0 μL was injected into a reversed-phase HPLC system.

#### HPLC Apparatus and Analytical Conditions

The HPLC system used in this study was manufactured by ESA Inc. (Chelmsford, MA, USA). The HPLC system was equipped with a pump (Model 582), an autosampler (Model 540), a UV detector (Model 526), and an integrator (CoulArray for Windows® [version 3.10]). Separation was performed using an Octa Decyl Silyl column (150.0 mm × 4.6 mm I.D., 3.0 μm) (Acclaim^TM^ 120; Thermo Fisher Scientific, Waltham, MA, USA) at 40°C. The detection wavelength was set at 220 nm. The mobile phase solvent consisted of acetonitrile and 60.6 mM potassium dihydrogen phosphate (45:55 v:v), including 880 mg/L sodium dodecyl sulfate, and was pumped at a flow rate of 0.5 mL/min. The assay was linear in the concentration range of 0.0–5.0 μg/mL, and the lowest detection concentration was 0.05 μg/mL.

### Statistical Analyses

Serum concentrations of ropivacaine and other variables are presented as mean ± standard deviation. EZR (Saitama Medical Center, Jichi Medical University, Saitama, Japan) and R (version 3.3.1) (2016-06-21, svn rev 70800, Vienna, Austria) were used for statistical analyses (14).

## Results

One patient was excluded due to sample failure; therefore, we analyzed a total of 30 patients. The patients' characteristics are shown in Table 1. The mean total dose of ropivacaine was 5.01 ± 0.68 mg/kg, with a maximum total dose of 6.30 mg/kg; the mean interval from T1 to T5 was 128.0 ± 17.7 min. Until the awake phase, there were no patients in whom the maximum concentration exceeded the toxicity threshold of 4.3 μg/mL (mean serum concentration: T1, 1.23 ± 0.36 μg/mL; T2, 1.09 ± 0.34 μg/mL; T3, 0.96 ± 0.28 μg/mL; T4, 0.83 ± 0.26 μg/mL; and T5, 0.82 ± 0.26 μg/mL).

**Table 1:** Patient data.

**Parameter**	**Value**
Age (years)	46.6 ± 10.8
Sex (male:female)	13:17
Height (cm)	162.1 ± 7.6
Weight (kg)	56.2 ± 9.4
Total dose of ropivacaine (mg/kg)	5.01 ± 0.68
Cases requiring additional scalp blocks	9

**Figure 1:**
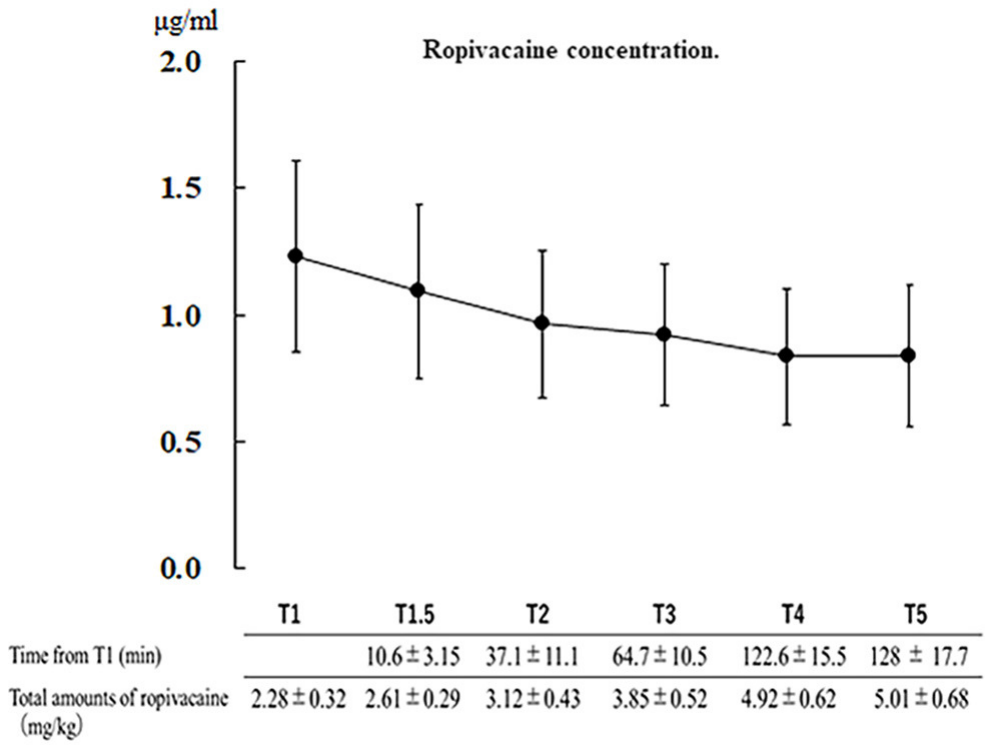
Serum concentration of ropivacaine at each point and injection time relative to the scalp block (T1). Data: mean ± standard deviation. T1.5, additional scalp block; T2, headpin site; T3, surgical site; T4, temporal muscle; and T5, dura mater.

No local anesthetic intoxication symptoms were observed during awakening in all cases. Twenty patients required additional administration of ropivacaine (Figure 1). Other complications in the awake phase were headache (*n* = 9, 30%), nausea (*n* = 4, 13%), seizure (*n* = 3, 10%), agitation (*n* = 2, 6.6%), and vomiting (*n* = 1, 3.3%). There were no cases of local anesthetic toxicity or desaturation events (Table 2).

**Table 2:** Incidence of intraoperative complications.

**Complication**	**Cases, *n* (%)**
Pain	9 (30.0)
Nausea	3 (10.0)
Vomiting	1 (3.0)
Seizure	3 (10.0)
Agitation	2 (6.6)
Airway complications (desaturation)	0 (0.0)

## Discussion

In AC, scalp blocks play a critical role in relieving intraoperative headache (5). Administering local anesthetic in the surgical field is effective (3) as there is a possibility of the analgesic effect being insufficient with the nerve block alone, and pain could manifest in the region where the nerve block is not effective. Therefore, local anesthetic agents should also be administered in other areas, such as the headpin site, skin, temporal muscle, and dura mater. Combined scalp blocks and administration of local anesthetic in the surgical field are key for successful AC (3, 4).

Our study revealed that, on average, 5.0 mg/kg of ropivacaine was repeatedly administered during the period of approximately 120 min before the awake phase, and none of the cases reached the intoxication level of 4.3 μg/mL. No patients presented with local anesthetic intoxication. Therefore, it is safe to administer ropivacaine at 5.0 mg/kg by the time of awakening in AC.

In this study, we collected arterial blood samples from patients to set the threshold for the systemic toxicity of ropivacaine. Knudsen et al. (12) reported 4.3 μg/mL of arterial ropivacaine as the threshold for systemic toxicity. Toju et al. (15) reported that the plasma concentration of ropivacaine at an earlier timepoint may be underestimated because arterial blood delivers the local anesthetic to the brain and heart where local anesthetic toxicity occurs. They also suggested that arterial plasma concentration may be better for analyzing the local anesthetic toxicity threshold.

In a previous study on patients who received ropivacaine and levobupivacaine during AC, blood concentrations were measured 15 min after injection. As per the report, 3.6 mg/kg of ropivacaine was safely used at the scalp blocks and headpin site (7). To our knowledge, that study was the first in which ropivacaine concentrations administered during AC were measured. Moreover, they reported that 2.5 mg/kg of levobupivacaine was safe for AC, with no episodes of local anesthetic intoxication (16). Our study revealed that higher doses of local anesthetic of up to 5.0 mg/kg can be administered repeatedly for AC, and there were no cases of local anesthetic intoxication.

Another study reported on the efficacy and safety of lidocaine and ropivacaine combinations used during AC (6). Combinations of local anesthetic agents have the potential to reduce efficacy and shorten the duration of anesthesia, as per a previous report (17). We, therefore, used ropivacaine as the only local anesthetic agent.

Several other studies on the acceptable safety of ropivacaine for other peripheral nerve blocks and local injections have been reported. These studies investigated the serum concentrations of ropivacaine by single or continuous injection, whereas we reported the safe dose of scalp blocks and local anesthetic agents after repeated injections. Hessian et al. (18) investigated plasma concentrations of ropivacaine in patients receiving postoperative transversus abdominis plane blocks and infusion for analgesia after laparotomy. They reported that four patients experienced neurological symptoms attributed to local anesthetic toxicity but did not have high plasma concentrations of ropivacaine, and that the estimated total amount of ropivacaine (3.6 mg) was a safe dose for transversus abdominis plane block. Koniuch et al. (19) reported that, during total knee arthroplasty, the total serum concentration of ropivacaine remained below the toxicity threshold after local infiltration analgesia using 270 mg ropivacaine with and without an additional 100 mg of perineural ropivacaine.

This study has several limitations. First, this was a single-arm study, so there was no control group. In AC, introducing local anesthetic agents, such as in scalp blocks, is essential for the successful operation (20), and it is ethically not possible to include a negative control group (no scalp blocks and no local anesthetic agents). If there are no scalp blocks, or if less local anesthetic was used for AC, the patient would suffer substantial intraoperative pain. Second, in this study, we measured the total plasma concentration only, whereas the toxicity of ropivacaine arises from the unbound fraction of ropivacaine (21), which was not measured owing to technical limitations.

In conclusion, our report suggests that repeated administration of a total dose of up to 5.0 mg/kg of ropivacaine have a possibility to be safe for AC, without eliciting addiction symptoms or elevating blood levels to the toxic range. Moreover, relatively large amounts of ropivacaine can be safely injected during AC.

## Data Availability Statement

The raw data supporting the conclusions of this article will be made available by the authors, without undue reservation.

## Ethics Statement

This study was approved by the UMIN Clinical Trial Registry (UMIN ID: 000030896; date of approval: January 19, 2018). The patients/participants provided their written informed consent to participate in this study. Written informed consent was obtained from the individual(s) for the publication of any potentially identifiable images or data included in this article.

## Author Contributions

TS, TA, and IA: anesthetic management of the patients. TS: writing—original draft preparation. AM: measurement of ropivacaine concentration. KM: operation on all cases. KM and KN: supervision of the drafting of the manuscript. All authors drafted and critically revised the manuscript and have read and approved the final manuscript.

## Conflict of Interest

The authors declare that the research was conducted in the absence of any commercial or financial relationships that could be construed as a potential conflict of interest.

## Publisher's Note

All claims expressed in this article are solely those of the authors and do not necessarily represent those of their affiliated organizations, or those of the publisher, the editors and the reviewers. Any product that may be evaluated in this article, or claim that may be made by its manufacturer, is not guaranteed or endorsed by the publisher.
